# Approximating the correction of weighted and unweighted orthology and paralogy relations

**DOI:** 10.1186/s13015-017-0096-x

**Published:** 2017-03-11

**Authors:** Riccardo Dondi, Manuel Lafond, Nadia El-Mabrouk

**Affiliations:** 1 Dipartimento di Lettere, Filosofia, Comunicazione, Università degli Studi di Bergamo, Via Donizetti 3, 24129 Bergamo, Italy; 2 Department of Mathematics and Statistics, University of Ottawa, Ottawa, Canada; 3 Département d’informatique et de recherche opérationnelle, Université de Montréal, Quebec, Canada

**Keywords:** Orthology, Paralogy, Approximation algorithms, Gene tree, Species tree

## Abstract

**Background:**

Given a gene family, the relations between genes (orthology/paralogy), are represented by a relation graph, where edges connect pairs of orthologous genes and “missing” edges represent paralogs. While a gene tree directly induces a relation graph, the converse is not always true. Indeed, a relation graph is not necessarily “satisfiable”, i.e. does not necessarily correspond to a gene tree. And even if that holds, it may not be “consistent”, i.e. the tree may not represent a true history in agreement with a species tree. Previous studies have addressed the problem of correcting a relation graph for satisfiability and consistency. Here we consider the weighted version of the problem, where a degree of confidence is assigned to each orthology or paralogy relation. We also consider a maximization variant of the unweighted version of the problem.

**Results:**

We provide complexity and algorithmic results for the approximation of the considered problems. We show that minimizing the correction of a weighted graph does not admit a constant factor approximation algorithm assuming the unique game conjecture, and we give an *n*-approximation algorithm, *n* being the number of vertices in the graph. We also provide polynomial time approximation schemes for the maximization variant for unweighted graphs.

**Conclusions:**

We provided complexity and algorithmic results for variants of the problem of correcting a relation graph for satisfiability and consistency. For the maximization variants we were able to design polynomial time approximation schemes, while for the weighted minimization variants we were able to provide the first inapproximability results.

## Background

Genes are the basic molecular units of heredity holding the information for producing all proteins required to build and maintain cells. They are the key for understanding genetic diversity, adaptation to environmental variation, drug resistance, and many other genetic features. Therefore, a first step of most genomic studies is to group genes into families. Gene families are usually inferred from sequence similarity, the underlying idea being that similar sequences reflect *homologous* genes that have diverged from a common ancestral sequence.

However, homology alone is not sufficient to decipher the properties of genes. Given a gene family, it is important to discriminate between two types of homologs: *orthologs* being gene copies originating from a speciation event, and *paralogs* originating from a duplication event. According to the orthology conjecture [1], orthologous genes are expected to be more similar in function than paralogs.

Various methods have been developed to discriminate between orthologous and paralogous genes. Tree-based methods consist in first constructing a phylogenetic tree for the gene family, and then, given a species tree, applying a reconciliation approach for inferring speciation and duplication nodes [2]. On the other hand, tree-free methods are based on gene clustering according to sequence similarity (c.f. for example the COG database [3], OrthoMCL [4], InParanoid [5], Proteinortho [6]), synteny [7, 8] or functional annotation of genes [9]. Results of these methods are pairwise orthology relations, or groups of orthologs, that can be represented as relation graphs, where vertices are genes and edges represent orthology relations between genes. Assuming a full inference of pairwise orthology relations, “missing” edges of the relation graph represent paralogy. In addition, as different inference methods may lead to different predictions, instead of a yes or no orthology assignment, existing methods can rather motivate a way of assigning a score to a given relation [10], leading to a weighted relation graph. For example, orthology predictions with OrthoMCL [4] are based on a weighted graph, where edge weights are related to the sequence similarity score of the adjacent genes, while InParanoid [5] provides a confidence value that shows how closely related a paralog is to its “seed ortholog”. Surprisingly, as far as we know, weighted orthology/paralogy relation graphs have not been formally considered in the literature.

While a gene tree induces a set of relations betwen genes, the converse is not always true, as a set of relations may or may not represent a valid history for the gene family. Two underlying questions are: (1) is the set of relations “satisfiable” i.e. is there a tree, with internal nodes labeled as duplication or speciation, containing them all? (2) is the set of relations “*S*-consistent” with the known species tree *S*, i.e. is there a tree containing the relations that is a “valid” gene tree “in agreement” with *S*? Polynomial-time algorithms exist for deciding satisfiability and *S*-consistency for a full [11–13] or partial [10] set of pairwise gene relations.

In this paper, we address both the weighted and unweighted variants of the full relation graph correction problem. First, for a full weighted relation graph *R*, we consider two minimization versions for the problem of correcting the graph by minimizing edit operations, i.e. adding or removing edges of minimum total weight, so that it represents a satisfiable or *S*-consistent set of relations. Then, we consider two maximization versions for the unweighted variant were we are given a full unweighted relation graph that has to be corrected with edit operations, so that the maximum number of relations is not modified.

In the unweighted case, the minimization variant of the satisfiability correction problem reduces to editing a minimum number of edges of *R* in order to make it $P_4$-free, which is known to be NP-hard [14]. In [13], an integer linear programming formulation is used to correct relation graphs of small size, which is also applicable to weighted graphs. In [15], the authors propose an approximation algorithm of factor $4 \Delta$, where $\Delta$ is the maximum degree of the input graph. The algorithm, however, offers no guarantees in the case of weighted graphs, as there are weighted instances on which the correction is arbitrarily far from optimal. It is shown in [16] that the minimum edge editing problem cannot be approximated within an “additive” factor of $n^{2 - \epsilon }$, for any $\epsilon > 0$. Yet, the authors give a class of polynomial time algorithms that are approximable within an additive factor of $\epsilon n^2$, for any $\epsilon > 0$. This implies a constant factor algorithm for graphs with an edit distance of $\Omega (n^2)$, but offers no guarantee in the other cases. Moreover, this algorithm only applies to unweighted graphs, and does not consider that two genes from the same species must remain paralogs. Finally in [14], parameterized versions of the algorithm are explored. As for the *S*-consistency correction problem, we proved in a previous paper [17] that it is NP-hard, which is the only result so far.

We show in, “Hardness of approximation of minimum weighted editing for satisfiability and consistency” section, that the weighted satisfiability and *S*-consistency problems are not approximable within a constant factor, assuming the unique games conjecture. We complement this result by showing in “A bounded approximation algorithm for minimum weighted editing for satisfiability and consistency” section that they can be approximated within a factor of *n* (the number of vertices of the relation graph). The maximization variants for unweighted graphs are then considered in “PTASs for maximum CoGraph editing and maximum consistency editing” section. We show that a result in [16] implies a polynomial time approximation scheme (PTAS) for satisfiability. Furthermore, we prove that, by applying more involved arguments, a PTAS also exists for the *S*-consistency problem. We conclude the paper with some open problems.

## Trees and orthology relations

A graph *H* is denoted $H = (V_H,E_H)$, where $V_H$ is its set of vertices (or *nodes* if *H* is a tree) and $E_H$ its set of edges. If *H* is a tree, degree one nodes are *leaves*.

### Trees

All considered trees are rooted and binary. Given a set *X*, a *tree T for X* is a tree whose leafset, which we denote by ${\mathcal{L}} (T)$, is in bijection with *X*. Given an internal node *u* of *T*, the subtree rooted at *u* is denoted $T_u$ and we call the leafset ${\mathcal{L}} (T_u)$ the *clade of* *u*. A node *u* is an *ancestor* of *v* if *u* is on the (inclusive) path between *v* and the root. If *u* and *v* are connected by an edge of *T*, then *v* is a *direct descendant* of *u*. We denote by *ch*(*u*) the set of direct descendants (children) of *u*. The *lowest common ancestor* (lca) of *u* and *v*, denoted $lca_T(u, v)$, is the ancestor common to both nodes that is the most distant from the root. We define $lca_T(U)$ analogously for a set $U \subseteq V(T)$.

A *species tree* *S* for a species set $\Sigma$ represents an ordered set of speciation events that have led to $\Sigma$: an internal node is an ancestral species at the moment of a speciation event, and its children are the new descendant species.

**Fig. 1:**
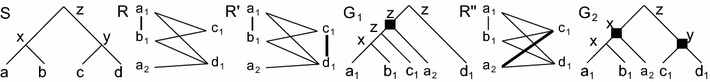
*S* is the species tree for $\Sigma = \{a,b,c,d\}$. The internal nodes, representing ancestral species, are labeled by *x*, *y* and *z*. *R* is a relation graph on gene set ${\Gamma }= \{a_1, a_2, b_1, c_1, d_1\}$. A gene name corresponds to the species it belongs to (e.g. $s(a_1) = a$). *R* is not satisfiable as the set of vertices $\{c_1, b_1, d_1, a_2\}$ induces a $P_4$. $R'$ is a satisfiable relation graph obtained from *R* by inserting the edge $\{c_1,d_1\}$, and $G_1$ is a *DS*-tree displaying every relation of $R'$ (each internal node *v* is labeled by $s_{G_1}(v)$). However, $G_1$ is not consistent with the species tree *S*. $R''$ is another correction of *R* that is *S*-consistent, as the tree $G_2$ displays the relations in $R''$ and is *S*-consistent. *Dup* nodes in *DS*-trees are marked by a *square*; all other nodes are speciation nodes

A *gene family* $\Gamma$ is a set of genes accompanied with a function $s : \Gamma \rightarrow \Sigma$ mapping each gene to its corresponding species. The evolutionary history of $\Gamma$ can be represented as a *node-labeled gene tree* for $\Gamma$, where each internal node refers to an ancestral gene at the moment of an event (either speciation or duplication), and is labeled as a speciation (*Spec*) or duplication (*Dup*) accordingly. Formally, we call a *DS-tree* for $\Gamma$ a pair $(G, ev_G)$, where *G* is a tree with ${\mathcal{L}} (G) = \Gamma$, and $ev_G:V_G \setminus {\mathcal{L}} (G) \rightarrow \{Dup, Spec\}$ is a function labeling each internal node of *G* as a duplication or a speciation. We may write *ev* instead of $ev_G$ when the context is clear. For example, in Fig. 1, $G_1$ and $G_2$ are two DS-trees.

According to the Fitch [18] terminology, we say that two genes *x*, *y* of $\Gamma$ are *orthologous in* *G* if $ev(lca_G(x, y)) = Spec$, and *paralogous in* *G* if $ev(lca_G(x, y)) = Dup$.

A *DS*-tree *G* for $\Gamma$ does not necessarily represent a valid history. For this to hold, any speciation node of *G* should reflect a clustering of species “in agreement” with *S* [10]. Formally *G* should be *S* *-consistent*, as defined below, where $s_G$ is the *LCA-mapping* function, mapping each gene, ancestral or extant, to a species as follows: if $g \in {\mathcal{L}} (G)$, then $s_G(g) = s(g)$; otherwise, $s_G(g) = lca_S( \{s(g') : g' \in {\mathcal{L}} (G_g) \})$.

#### **Definition 1**

Let *S* be a species tree and *G* be a *DS*-tree. Let *v* be an internal node of *G* such that $ev(v) = Spec$. Then the speciation node *v*, with children $v_1$ and $v_2$, is *S* *-consistent* iff none of $s_G(v_1)$ and $s_G(v_2)$ is an ancestor of the other. We say that *G* is *S* *-consistent* iff every speciation node of *G* is *S*-consistent.

For example, in Fig. 1, $G_1$ is not *S*-consistent as the root of $G_1$ is not *S*-consistent.

### Relation graphs

For a graph $H=(V_H,E_H)$, we denote the complementary set of $E_H$ by $\overline{E_H}= \{ \{ u,v \}: u,v \in V_H, \{ u,v \} \notin E_H\}$. Let $V'$ be a subset of $V_H$. The *subgraph of* *H* *induced by* $V'$, denoted $H[V']$, is the subgraph of *H* with vertex-set $V'$ having every edge $\{u,v\}$ of *H* for $u, v \in V'$. If *I* is another graph, we say *H* is *I-free* if there is no $V'\subseteq V_H$ such that $H[V']$ is isomorphic to *I*.

A *relation graph* *R* on a gene family $\Gamma$ is a graph with vertex set $V_R = \Gamma$, in which we interpret each edge $\{u,v\}$ of $E_R$ as an orthology relation between *u* and *v*, and each “missing” edge $\{u,v\} \in \overline{E_R}$, also called *non-edge*, as a paralogy relation. Notice that if $s(u) = s(v)$, then $\{u,v\}$ must be a non-edge (*u* and *v* are paralogous). We denote $n = |V_R|$.

A *DS*-tree *G* leads to a relation graph, denoted *R*(*G*), with vertex set ${\mathcal{L}} (G)$ and edge set corresponding to all gene pairs that are orthologous in *G*. Conversely, a relation graph *R* does not necessarily lead to a *DS*-tree. If this is the case, i.e. if there is a *DS*-tree *G* such that $R(G) = R$, then *R* is said *satisfiable*. As shown in [12], a relation graph *R* is satisfiable if and only if *R* is $P_4$-free, meaning that, for any four vertices of *R*, the induced graph is not a path of length 3 (number of edges). The $P_4$-free graphs are sometimes called *cographs*. See Fig. 1 for an example.

As a *DS*-tree does not necessarily represent a true history for $\Gamma$, satisfiability of a relation graph does not ensure a possible translation in terms of a history for $\Gamma$. For this to hold, *R* should also be *consistent* with the species tree, according to the following definition.

#### **Definition 2**

Let *S* be a species tree. A relation graph *R* for $\Gamma$ is *S*-consistent if and only if *R* is satisfiable by a *DS*-tree *G* which is itself *S*-consistent.

### Problem statements

We call a *weight* for a relation graph $R=(V_R,E_R)$ a function $w : {V_R \atopwithdelims ()2} \rightarrow \mathbb {R}^+$ on its vertex pairs. Notice that *w* assigns a weight to both edges (orthologies) and non-edges (paralogies). We shall assume that if $s(u) = s(v)$ for two genes *u* and *v*, then $\{u, v\} \in \overline{E_R}$ and $w(\{u, v\}) = \infty$. The weight function *w* is extended to any $I_R \subseteq {V_R \atopwithdelims ()2}$ by defining $w(I_R) = \sum _{\{x,y\} \in I_R} w(\{x,y\})$.

Given a relation graph $R=(V_R,E_R)$, an *edge-editing* of *R* is a pair $E_R^* = (E_R^+, E_R^-)$ with $E_R^+ \subseteq \overline{E_R}$ and $E_R^- \subseteq E_R$. We denote by $R(E_R^*)$ the graph $R(E_R^*)=(V_R, (E_R \cup E_R^+) \setminus E_R^-)$. In other words, $E_R^+$ (respectively $E_R^-$) denotes inserted (respec. removed) edges. Given a relation graph $R' =(V_{R'},E_{R'})$ computed from *R* by edge insertion and removal, the set of removed edges is $E_R^-=E_R\setminus E_{R'}$, and the set of inserted edges is $E_R^+=E_{R'}\setminus E_R$. For example, for the graph $R'$ of Fig. 1, $E^+_R = \{\{c_1,d_1\}\}$ and $E^-_R = \emptyset$. An *edge-editing* $E_R^*$ *is said* $P_4$ *-free* if $R(E_R^*)$ is itself $P_4$-free.

The problems considered in “Hardness of approximation of minimum weighted editing for satisfiability and consistency” section and “A bounded approximation algorithm for minimum weighted editing for satisfiability and consistency” section are the following. The first problem asks for a satisfiable relation graph, hence no species tree is considered, while the second asks for an *S*-consistent relation graph, hence the input contains also a species tree.

#### *Minimum weighted editing for satisfiability (MinWES)*

Input: A relation graph $R=(V_R,E_R)$ and a weight function *w*;
Output: A satisfiable relation graph $R'=(V_{R},E_{R'})$, obtained from *R* by an edge-editing $E_R^* = (E_R^+, E_R^-)$ that minimizes $w(E_R^+) + w(E_R^-)$.

#### *Minimum weighted editing for consistency (MinWEC)*

Input: A relation graph $R=(V_R,E_R)$, a weight function *w* and a species tree *S* for $\Sigma$ (the set of species containing the genes represented by *R*);
Output: An *S*-consistent relation graph $R'=(V_{R},E_{R'})$, obtained from *R* by an edge-editing $E_R^* = (E_R^+, E_R^-)$ that minimizes $w(E_R^+) + w(E_R^-)$.

Below is a formal statement of the corresponding maximization version of MinWES for unweighted graphs, considered in “PTASs for maximum CoGraph editing and maximum consistency editing” section. Remember that edges represent orthologies, while non-edges are paralogies. Maximizing conservation therefore requires accounting for both edges and non-edges.

#### *Maximum editing for satisfiability (MaxES)*

Input: A relation graph $R=(V_R,E_R)$;
Output: A satisfiable relation graph $R'=(V_{R},E_{R'})$ obtained from *R* by an edge-editing, such that its *value* $|E_R\cap E_{R'}| + |(\overline{E_R}\cap \overline{E_{R'}})|$ is maximized.

#### *Maximum editing for consistency (MaxEC)*

Input: A relation graph $R=(V_R,E_R)$ for a gene family with genes belonging to genomes in $\Sigma$, a species tree *S* for $\Sigma$;
Output: An *S*-consistent relation graph $R'=(V_{R},E_{R'})$ obtained from *R* by an edge-editing, such that its *value* $|E_R\cap E_{R'}| + |(\overline{E_R}\cap \overline{E_{R'}})|$ is maximized.

## Hardness of approximation of minimum weighted editing for satisfiability and consistency

We show that MinWES is unlikely to be approximable within a constant factor, by presenting a gap-preserving reduction from Minimum Multi-Cut. First, we consider the variant of MinWES, called Minimum Weighted Removal for Satisfiability (MinWRS), where only edge removal is allowed, then we easily extend the result to MinWES.

Given a graph $H=(V_H,E_H)$, and a set $X \subseteq {V_H \atopwithdelims ()2}$ (i.e. a set of pairs), Minimum Multi-Cut asks for a set $E'_H$ of minimum cardinality such that each pair $\{v_i,v_j\} \in X$ is disconnected in $H'=(V_H, E_H \setminus E'_H)$.

Given an instance $H=(V_H,E_H,X)$ of Minimum Multi-Cut, we construct an instance $R=(V_R,E_R,w)$ of MinWRS as follows. The vertex set $V_R$ includes, for each $v_i \in V_H$, two vertices $v_{i,R}$ and $v'_{i,R}$. That is, $V_R = \{ v_{i,R}, v'_{i,R}: v_i \in V_H \}$.

For any distinct $x, y \in V_R$, we set $s(x) \ne s(y)$, and hence there are no “forced” paralogs. As for $E_R$, it is defined as follows, where $q = |V_H|^5+1$.

- For each $v_i \in V_H$, define an edge $\{ v_{i,R}, v'_{i,R} \}$ in $E_R$ of weight $$q'= q |E_H| + 2\left( \left( {\begin{array}{c}|V_H|\\ 2\end{array}}\right) - |E_H| \right)$$;
- For each $\{v_i,v_j\} \in X$, define an edge $\{ v_{i,R}, v_{j,R} \}$ in $E_R$ with weight *q* if $\{ v_i,v_j \} \in E_H$, and with weight 1 if $\{ v_i,v_j \} \notin E_H$;
- For each $\{v_i,v_j\} \notin X$, define the edges $\{ v_{i,R}, v'_{j,R} \}$ and $\{ v'_{i,R}, v_{j,R} \}$ in $E_R$, each with weight *q* / 2 if $\{ v_i,v_j \} \in E_H$, and with weight 1 if $\{ v_i,v_j \} \notin E_H$.

For each $\{ u_R,v_R \} \in \overline{E_R}$, $\{ u_R,v_R \}$ has weight $q'$. Notice however, that, since edge insertion is not allowed in Minimum Weighted Co-Graph Deletion, the weight of $\{ u_R,v_R \}$ never contributes to the cost of a solution of Minimum Weighted Co-Graph Deletion.

We first show that there is a correspondance between solutions to the two problems on our constructed instances.

We first bound the number of edges of weight 1 in *R*.

### **Claim 1**

*Let* $H=(V_H,E_H,X)$ *be an instance of* *Minimum Multi-Cut* *and let* $R=(V_R,E_R,w)$ *be the corresponding instance of* *Minimum Weighted Co-Graph Deletion* *. Then,* *R* *contains at most* $$2 \left( \left( {\begin{array}{c}|V|\\ 2\end{array}}\right) - |E_H|\right)$$ *edges of weight 1.*

### *Proof*

Consider the edges connecting vertices $v_{i,R}$ and $v_{j,R}$; $v_{i,R}$ and $v_{j,R}$ are connected by an edge of weight 1 if and only if $\{v_i,v_j\} \notin E_H$ and $\{v_i,v_j\} \in X$.

Consider the edges connecting vertices $v_{i,R}$ and $v'_{j,R}$, $v'_{i,R}$ and $v_{j,R}$. $v_{i,R}$, $v'_{j,R}$ (and $v'_{i,R}$,$v_{j,R}$) are connected by an edge of weight 1 if $\{ v_i,v_j \} \notin E_H$ and $\{v_i,v_j\} \notin X$.

Any other edge has weight >1, hence the lemma follows. $\square$

Now, we present the main results needed to prove the inapproximability of Minimum Weighted Co-Graph Deletion.

### **Lemma 1**

*Let* $H=(V_H,E_H,X)$ *be an instance of* *Minimum Multi-Cut* *and let* $R=(V_R,E_R,w)$ *be the corresponding instance of* *Minimum Weighted Co-Graph Deletion* *. Given a solution* $E'_H$ *of* *Minimum Multi-Cut* *, we can compute in polynomial time a solution of* *Minimum Weighted Co-Graph Deletion* *of weight at most* $$q|E'_H|+ 2\left( \left( {\begin{array}{c}|V_H|\\ 2\end{array}}\right) - |E_H|\right)$$.

### *Proof*

Given a set $E'$ that defines a multicut in *H*, let $V_{H,1}, \dots , V_{H,p}$ be the sets of vertices of the connected components in the graph $V'_H=(V'_H, E_H \setminus E'_H)$.

We define a solution of Minimum Weighted Co-Graph Deletion over instance *R* as follows. We construct the partition $V_{R,1}, \dots , V_{R,p}$ of the vertices of *R* such that $v_{j,R}$ and $v'_{j,R}$ belong to set $V_{R,i}$ if and only if $v_j \in V_{H,i}$. All edges having their endpoints in two distinct $V_{R, i}, V_{R, j}$ are removed.

We claim that the computed graph $R'$ induced by the partition is $P_4$-free. By construction, for each $v_{j,R}$, $v'_{j,R}$, $v_{h,R}$, $v'_{h,R}$ that belong to $V_{R,i}$, the edges $\{ v_{j,R}, v'_{h,R} \}$ and $\{ v'_{j,R}, v_{h,R} \}$ belong to $E_R$ (because $\{v_j, v_h\} \notin X$). Moreover, there is no edge between $v_{j, R}$ and $v_{h, R}$, nor between $v'_{j, R}$ and $v'_{h, R}$. Thus any path on four vertices in the graph on vertex set $V_{i, R}$ must be either of the form $v_{j,R}v'_{h, R}v_{k, R}v'_{{\ell }, R}$, or of the form $v'_{j,R}v_{h, R}v'_{k, R}v_{{\ell }, R}$. In both cases, the endpoints of the path share an edge, and thus cannot induce a $P_4$.

Now, consider the edges $\{v_i,v_j\} \in E'_H$. If $\{v_i,v_j\} \in X$, the corresponding solution of Minimum Weighted Co-Graph Deletion removes an edge of weight *q*, namely $\{ v_{i,R}, v_{j,R} \}$. If $\{v_i,v_j\} \notin X$, the corresponding solution of Minimum Weighted Co-Graph Deletion removes two edges of weight *q* / 2, namely $\{ v_{i,R}, v'_{j,R} \}$ and $\{ v'_{i,R}, v_{j,R} \}$. Hence those edges have a total weight $q|E'_H|$. Since at most $$2 \left( \left( {\begin{array}{c}|V_H|\\ 2\end{array}}\right) - |E_H| \right)$$ edges of weight 1 are removed (see Claim 1), we can conclude that the lemma holds. $\square$

### **Lemma 2**

*Let* $H=(V_H,E_H,X)$ *be an instance of* *Minimum Multi-Cut* *and let* $R=(V_R,E_R,w)$ *be the corresponding instance of* *Minimum Weighted Co-Graph Deletion* *. Given a solution* $R'$ *of* *Minimum Weighted Co-Graph Deletion* *of weight at most* $$qW+2\left( \left( {\begin{array}{c}|V_H|\\ 2\end{array}}\right) - |E_H|\right)$$ *for some integer* *W, we can compute in polynomial time a multicut* $E'_H$ *of H of size at most W*.

### *Proof*

Consider a solution $R'=(V_R,E'_R,w)$ of Minimum Weighted Co-Graph Deletion over instance $R=(V_R,E_R,w)$ of weight at most $$qW+2\left( \left( {\begin{array}{c}|V_H|\\ 2\end{array}}\right) - |E_H|\right)$$, with $W\le |E_H|$. First, notice that no edge $\{v_{i,R}, v'_{i,R} \}$, with $1 \le i \le |V|$, is removed to obtain $R'$, since the weight of such an edge is greater than $$qW+2\left( \left( {\begin{array}{c}|V_H|\\ 2\end{array}}\right) - |E_H|\right)$$.

Consider now two vertices $v'_{i,R}$, $v'_{j,R}$, such that, given the corresponding vertices $v_i$, $v_j$ in *H*, we have $\{v_i,v_j\} \in X$. By construction there is a $P_4$ in *R*, namely $v'_{i,R}, v_{i,R}, v_{j,R}, v'_{j,R}$. It follows that the edge $\{v_{i,R}, v_{j, R}\}$ must be removed in $R'$. Moreover, we claim that in $R'$, the vertices $v'_{i,R}$, $v'_{j,R}$ must be disconnected. Assume by contradiction that this does not hold, and that $v'_{i,R}$, $v'_{j,R}$ belong to the same connected component of $R'$. Consider the shortest path *P* that connects vertices $v_{i,R}$ and $v_{j,R}$ in $R'$. Then *P* has length at least 2. Note that as *P* is a shortest path, it has no chord, i.e. non-consecutive vertices of *P* cannot share an edge.

Suppose that *P* does not include the vertex $v'_{i,R}$. Then we can assume that $v_{i,R}$ is adjacent in *P* to a vertex $v'_{t,R}$, since if it is adjacent to a vertex $v_{q,R}$, then the vertices $v_{i,R}$, $v'_{i,R}$, $v_{q,R}$, and $v'_{q,R}$ would induce a $P_4$. Now, if $v'_{t,R}$ is adjacent to $v_{j,R}$, then $v'_{i,R}$, $v_{i,R}$, $v'_{t,R}$ and $v_{j,R}$ induce a $P_4$. If there is no such $v'_{t,R}$, then *P* has length at least 3 and it must therefore contain an induced $P_4$.

So suppose instead that *P* includes the vertex $v'_{i,R}$. Since by construction $v'_{i,R}$ is not adjacent to $v_{j,R}$ and it is not adjacent to any $v'_{t,R}$, with $t \ne i$, while it is adjacent to $v_{i,R}$, *P* has length at least 3, and again must have an induced $P_4$.

We can conclude that when $\{v_i,v_j\} \in X$, the corresponding vertices $v'_{i,R}$, $v'_{j,R}$ belong to disconnected connected components of $R'$. Hence we can compute a multi-cut of *H* as follows:

$$\begin{aligned} E'_H &= \{ \{ v_i,v_j \}: \{v_{i,R} , v_{j,R}\}, {\text { of weight}}\,{q}, {\text { or }} \{v_{i,R} , v'_{j,R} \}, \{v'_{i,R} , v_{j,R} \} {\text {, of weight}}\,{\frac{q}{2},} \\ & \quad {\text {are removed in }}{R' }.\} \end{aligned}$$

$E'_H$ is a multi-cut, since each $\{v_i,v_j\} \in X$ is disconnected. Now, recall that $R'$ is obtained by removing edges of overall weight at most $$qW+2\left( \left( {\begin{array}{c}|V_H|\\ 2\end{array}}\right) - |E_H|\right)$$. Since edge edge in $E'_H$ corresponds to edges of overall weight *q* in *R* (an edge $\{ v_{i,R}, v_{j,R} \}$ of weight *q* if $\{v_{i}, v_{j}\} \in X$, or two edges of weight *q* / 2, namely $\{ v_{i,R}, v'_{j,R} \}$ and $\{ v'_{i,R}, v_{j,R} \}$ if $\{v_i,v_j\} \notin X$), we must have $|E'_H| \le W$. $\square$

Assuming the unique games conjecture, the proof of inapproximability of Minimum Weighted Co-Graph Deletion is deduced from the inapproximability of Minimum Multi-Cut [19].

### **Theorem 1**

*Minimum Weighted Co-Graph Deletion* *is not approximable within a constant factor assuming the unique games conjecture.*

### *Proof*

Given a graph *H* instance of Minimum Multi-Cut and the corresponding instance *R* of Minimum Weighted Co-Graph Deletion, denote by $OPT_M$ ($AP_M$, respectively) the value of an optimal solution (of an approximation solution, respectively) of Minimum Multi-Cut on instance *H*, and denote by $OPT_C$ ($AP_C$, respectively) the value of an optimal solution (of an approximation solution, respectively) of Minimum Weighted Co-Graph Deletion on instance *R*. Define $$z= 2\left( \left( {\begin{array}{c}|V_H|\\ 2\end{array}}\right) - |E_H|\right)$$. By Lemma 2, we assume that $AP_C(R) \ge AP_M(H) q$, as there exists an algorithm that given a solution of Minimum Weighted Co-Graph Deletion of value $AP_C(R)$ computes in polynomial time a solution of Minimum Multi-Cut having value at most $AP_M(H)$ with $AP_M(H) q \le AP_M(H) q + z \le AP_C(R)$. Also, by Lemma 1, we have $OPT_C(R) \le OPT_M(H)q + z$, as for any optimal solution of Minimum Multi-Cut of value $OPT_M(H)q$, there is an algorithm that computes in polynomial time a solution of Minimum Weighted Co-Graph Deletion having value $OPT_C(R)$ with $OPT_C(R) \le OPT_M(H)q + z$.

We have that

$$\begin{aligned} \frac{AP_C(R)}{OPT_C(R)} \ge \frac{AP_M(H)q}{OPT_M(H)q+z} &= \frac{AP_M(H)q+AP_M(H)z - AP_M(H)z}{OPT_M(H)q+z}\\ &= \frac{AP_M(H)q+AP_M(H)z}{OPT_M(H)q+z} - \frac{AP_M(H)z}{OPT_M(H)q+z}\\ &\ge \frac{AP_M(H)q+AP_M(H)z}{OPT_M(H)q+OPT_M(H)z} - \frac{AP_M(H)z}{OPT_M(H)q+z}\\ &= \frac{AP_M(H)(q+z)}{OPT_M(H)(q+z)} - \frac{AP_M(H)z}{OPT_M(H)q+z}\\ &=\frac{AP_M(H)}{OPT_M(H)} - \frac{AP_M(H)z}{OPT_M(H)q+z} \end{aligned}$$

where we assume $OPT_M(H) \ge 1$ for the second inequality (the case $OPT_M(H) = 0$ can be checked in polynomial time). Since Minimum Multi-Cut is not approximable within a constant factor assuming the unique games conjecture [19], even on unweighted graphs, it follows that

$$\begin{aligned} \frac{AP_M(H)}{OPT_M(H)} \ge \alpha \end{aligned}$$

on an infinity of instances of *H* for any constant $\alpha \ge 1$. As a consequence, for any constant $\alpha \ge 1$, an infinity of instances of *R* yield:

$$\begin{aligned} \frac{AP_C(R)}{OPT_C(R)} \ge \alpha -\frac{AP_M(H)z}{OPT_M(H)q+z} \end{aligned}$$

Since $q=n^5+1$, $AP_M(H) \le n^2$ and $z \le n^2$, it follows that $\frac{AP_M(H)z}{OPT_M(H)q+z} \le 1/n$. Combining the last two inequalities, we have that

$$\begin{aligned} \frac{AP_C(R)}{OPT_C(R)} \ge \alpha - 1/n \ge \beta \end{aligned}$$

for any constant $\beta \ge 1$, which concludes the proof. $\square$

The result of Theorem 1 can be easily extended to Minimum Weighted Co-Graph Editing.

### **Corollary 1**

*Minimum Weighted Co-Graph Editing* *is not approximable within a constant factor assuming the unique games conjecture.*

### *Proof*

The result follows by a gap-preserving reduction similar to that for Minimum Weighted Co-Graph Deletion. Recall that for each pair $\{ u_R,v_R \} \in \overline{E_R}$, a weight of $q'$ is associated with $\{ u_R,v_R \}$. Consider a solution $R'$ of Minimum Weighted Co-Graph Editing on instance *R* that has cost not greater than $$qW+\left( \left( {\begin{array}{c}|V_H|\\ 2\end{array}}\right) - |E_H|\right)$$ $$+\left( {\begin{array}{c}|V_H|\\ 2\end{array}}\right)$$. It is easy to see that $R'$ is obtained without any edge insertion. $\square$

The inapproximability result for Minimum Weighted Co-Graph Editing is easily extended to MinWEC. This is achieved by defining a species tree *S* on $V_R$ such that the root of *S* is connected to two subtrees, one with leafset $\{v_{i,R}:v_i \in V_H \}$, one with leafset $\{v'_{i,R}:v_i \in V_H \}$, and showing that any solution to our instance of Minimum Weighted Co-Graph Deletion must agree with this species tree.

### **Corollary 2**

*MinWEC* *is not approximable within a constant factor assuming the unique games conjecture.*

### *Proof*

The result follows by a gap-preserving reduction similar to that for Minimum Weighted Co-Graph Deletion and Minimum Weighted Co-Graph Editing. Define a species tree *S* on $V_R$ such that the root of *S* is connected to two subtrees, one with leafset $\{v_{i,R}:v_i \in V_H \}$, one with leafset $\{v'_{i,R}:v_i \in V_H \}$.

Consider the partition $V_{R,1}, \dots , V_{R,p}$ of the vertices of a solution $R'$ of Minimum Weighted Co-Graph Deletion and Minimum Weighted Co-Graph Editing. Each connected component $V_{R,t}$ that contains vertices $v_{i,R}$, $v'_{i,R}$, $v_{j,R}$, $v'_{j,R}$, contains only edges $\{v_{i,R},v'_{i,R}\}$, $\{v_{j,R},v'_{j,R}\}$, $\{v_{i,R},v'_{j,R}\}$, $\{v_{j,R},v'_{i,R}\}$.

For each set $V_{R,i}$, we construct a tree $G_{R,i}$ by defining two subtrees $G^1_{R,i}$ and $G^2_{R,i}$ such that $G^1_{R,i}$ has leafset $\{v_{j,R}: v_{j,R} \in V_{R,i} \}$ and $G^2_{R,i}$ has leafset $\{v'_{j,R}: v'_{j,R} \in V_{R,i} \}$. Each node of $G^1_{R,i}$ and $G^2_{R,i}$ is associated with a duplication. $G_{R,i}$ is obtained by joining $G^1_{R,i}$ and $G^2_{R,i}$ in a root, associated with a speciation. Finally, the subtrees $G_{R,1}, \dots , G_{R,p}$ are joined in a gene tree *G* by duplication nodes (with any topology). By construction, *G* is *S*-consistent, thus the hardness result can be extended to MinWEC. $\square$

## A bounded approximation algorithm for minimum weighted editing for satisfiability and consistency

While MinWES and MinWEC are not approximable within a constant factor, we show here that they can be approximated within factor $n = |V(R)|$, and we give the corresponding algorithms. Despite being a large approximation factor, this is the best known bound so far and shows that the problems have polynomially bounded approximability. We first describe the approximation algorithm for MinWES.

Denote by $\overline{R}=(V_R, \overline{E_R})$ the *complement* of the graph $R = (V_R,E_R)$. A well-known property of cographs is given by the following lemma.

### **Lemma 3**

 [20] *A graph* *R is* $P_4$ *-free if and only if for any* $X \subseteq V_R$ *, one of R*[*X*] *or* $\overline{R[X]}$ *is disconnected.*

This motivates a greedy min-cut approach for MinWES, performing an edge-editing of minimum weight disconnecting the graph or its complement, and iterating recursively on the resulting components. This is the main idea of Algorithm MinCut-Cograph-Editing below. Note that assuming forced paralogs have infinite weight, this algorithm will never make two genes from the same species orthologs.

More formally, let $R=(V_R,E_R)$ be a relation graph accompanied with a weight function *w*. Define a *cut* $C = \{X, Y\}$ as a partition of $V_R$ with *X* and *Y* being non-empty sets, and denote $E_R(C) = \{ \{x,y\} \in E_R : x \in X, y \in Y\}$. The weight of *C* is $w(C) = w(E_R(C))$. The cut *C* is a *minimum cut* or *MinCut* if no other cut has a smaller weight *w*(*C*). *Applying a cut* *C* to *R* consists in removing all edges of $E_R(C)$ from *R*.

*Complexity:* A MinCut of a given graph of *n* vertices and *m* edges can be found in time $O(nm+n^2\log n)$ using the Stoer–Wagner algorithm [21]. In the MinCut-Cograph-Editing algorithm, MinCut is applied to both *R* and $\overline{R}$. As at least one of these two graphs has $\Omega (n^2)$ edges, the required time for MinCut is therefore $O(n^3)$. This step is repeated at most *n* times, hence the overall time complexity of MinCut-Cograph-Editing is $O(n^4)$.

The remaining of this section is dedicated to proving Theorem 2, which states that MinCut-Cograph-Editing is an *n*-approximation algorithm. We denote by $\sigma _R$ the minimum weight of a $P_4$-free edge-editing of *R*. If $X \subseteq V_R$, we denote $\sigma _{R[X]}$ by $\sigma _X$.

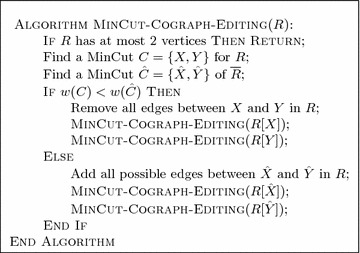



### **Lemma 4**

*Let C be a minimum cut of R, and let* $\hat{C}$ *be a minimum cut of* $\overline{R}$ *. Then* $\sigma _R \ge \min \{w(C), w(\hat{C})\}$.

### *Proof*

Let $E_R^*$ be a $P_4$-free edge-editing of *R*. By Lemma 3, either $R(E_R^*)$ or its complement is disconnected, implying that $E_R^*$ must apply some cut on either *R* or $\overline{R}$. This cut is at best a minimum cut. $\square$

### **Lemma 5**

*Let* $\{X, Y\}$ *be a partition of V. Then,* $\sigma _R \ge \sigma _X + \sigma _Y$.

### *Proof*

Let $E_R^*$ be a $P_4$-free edge-editing of weight $\sigma _R$, and let $R' = R(E_R^*)$. Assume that $E_R^*$ has a weight stricly smaller than $\sigma _X + \sigma _Y$. Then, since $R'[X]$ and $R'[Y]$ are $P_4$-free, there must either be an edge-editing of *R*[*X*] of weight smaller than $\sigma _X$, or an edge-editing of *R*[*Y*] of weight smaller than $\sigma _Y$, contradicting the definition of $\sigma _X$ and $\sigma _Y$. $\square$

### **Theorem 2**

MinCut-Cograph-Editing *is an n factor approximation algorithm for* MinWES.

### *Proof*

Denote by $\beta (R)$ the weight of the edge-editing found by the algorithm on *R*. We proceed by induction on $n = |V_R|$ to show that $\beta (R) \le n\sigma _R$. The statement is trivial for $n \le 3$ (as there is nothing to correct), so assume that the algorithm finds a solution of weight $\beta (R) \le k\sigma _R$ for any graph of size at most $k < n$. The algorithm applies a minimum cut $C = \{X, Y\}$ on *R* or $\overline{R}$, and proceeds recursively on *X* and *Y*, with $|X|, |Y| \le n - 1$. By the induction hypothesis, we have

$$\begin{aligned}\beta (R)&\le |X|\sigma _X + |Y|\sigma _Y + w(C) \le (n - 1)(\sigma _X + \sigma _Y) + w(C) \\&\le (n - 1)\sigma _R + \sigma _R = n \sigma _R \end{aligned}$$

where the last inequality holds due to Lemmas 4 and  5. $\square$

**Fig. 2:**
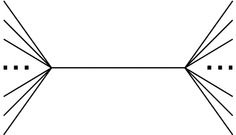
A graph *R* with all edges of weight 1

It is possible to show that the approximation factor of MinCut-Cograph-Editing is tight, as shown in Fig. 2. Suppose all weights are equal to one. Clearly, an optimal solution of weight 1 is obtained by removing the middle edge. However, a minimum cut $\{X, Y\}$ can be found by taking *X* as a single vertex of degree one, and *Y* as the rest. In this manner, the algorithm might remove up to $n - 3$ edges before *H* becomes $P_4$-free, which is $n - 3$ times worse than optimal.

Notice however that a solution of MinCut-Cograph-Editing on the example of Fig. 2 cannot be $2\Delta (H)$ times worse than the optimal solution, where $\Delta (H)$ is the degree of *H* (by putting half the leaves left and the other half right). We do not know whether MinCut-Cograph-Editing offers any guarantee in relation to $\Delta (H)$ or $\Delta (\overline{H})$.

By modifying MinCut-Cograph-Editing, it is possible to design an *n* factor approximation algorithm for MinWEC. The main difference with respect to MinCut-Cograph-Editing, is that the algorithm considers a minimum cut on a subset of *R* and a cut on a subset of $\overline{R}$ induced by the species tree *S*.

We first provide the detailed MinCut-Cograph-Editing-Cons algorithm, and show that it also is a *n*-factor approximation. Given a species tree *S* and a set $Z \subseteq V_R$, let $\Sigma (Z)=\{ s(x):x \in Z \}$. Let $S|\Sigma (Z)$ be the subtree of *S* restricted to $\Sigma (Z)$ and let $X_S$, $Y_S$ be the clades of the left and right child, respectively, of the root of $S|\Sigma (Z)$. Consider the sets $X=\{ x:s(x) \in X_S \}$ and $Y=\{ y:s(y) \in Y_S \}$, the cut $C_S(Z)$ on $\overline{R}[Z]$ is defined as $C_S(Z)=\{ X_R,Y_R \}$. Observe that $C_S(Z)$ is the only possible cut on $\overline{R}$ that maintains *S*-consistency, as this cut corresponds to a speciation in a *DS*-tree, and speciations must separate genes according to *S*. Therefore, it suffices to modify MinCut-Cograph-Editing by forcing the cut $\hat{C}$ to be $C_S(Z)$. Call this modified algorithm MinCut-Cograph-Editing-Cons.

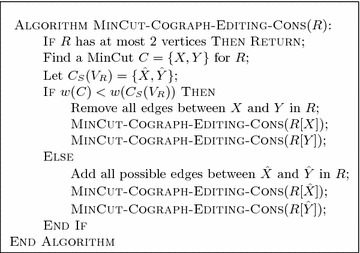



### **Theorem 3**

MinCut-Cograph-Editing-Cons *is an n factor approximation algorithm for* MinWEC.

### *Proof*

Denote by $\beta (R)$ the weight of the edge-editing found by the algorithm on *R*. We proceed by induction on $n = |V_R|$ to show that $\beta (R) \le n\sigma _R$. The statement is trivial for $n \le 2$ (as there is nothing to correct), so assume that the algorithm finds a solution of weight $\beta (R) \le k\sigma _R$ for any graph of size at most $k < n$.

The algorithm applies a cut $C = \{X, Y\}$ which is either a minimum cut on *R* or it is the cut $C_S(V_R)$, and proceeds recursively on *X* and *Y*, with $|X|, |Y| \le n - 1$. By the induction hypothesis, we have

$$\begin{aligned} \beta (R)&\le |X|\sigma _X + |Y|\sigma _Y + w(C) \le (n - 1)(\sigma _X + \sigma _Y) + w(C) \end{aligned}$$

Now, similarly to Lemma 4, we have that $w(C) \le \sigma _R$. First, let $G'$ be the gene tree associated with a solution of MinWEC over instance *R*. If *C* is a minimum cut on *R*, it holds due to the proof Lemma 4. If *C* is $C_S(V_R)$, then notice that, in order to guarantee the consistency with *S*, the root of $G'$ must induce exactly the cut $C_S(V_R)$.

Lemma 5 holds also for MinWEC, hence

$$\begin{aligned} \beta (R)&\le |X|\sigma _X + |Y|\sigma _Y + w(C) \le (n - 1)(\sigma _X + \sigma _Y) + w(C) \\&\le (n - 1)\sigma _R + \sigma _R = n \sigma _R \end{aligned}$$

thus concluding the proof. $\square$

## PTASs for maximum CoGraph editing and maximum consistency editing

In this section, we consider the MaxES and the MaxEC problems. Although sharing the same objectives, the minimization and maximization variants are not equivalent from an approximation point of view.

Given a relation graph *R*, the value of a solution $R'$ for MaxES (MaxEC, respectively) over instance *R* [over instance (*R*, *S*), respectively] is called the *agreement* value of $R'$ and it is denoted by $A(R',R)$. Moreover, given a gene tree *G*, we denote by *A*(*G*, *R*) the agreement between the relation graph associated with *G* and *R*.

Next, we give a bound on the agreement value returned by an optimal solution of MaxES and MaxEC.

### **Lemma 6**

*Given a relation graph R (a relation graph R and a species tree S, respectively), an optimal solution of* *MaxES* *over instance R [an optimal solution of* *MaxEC* *over instance (R, S), respectively] has an agreement value of at least* $\frac{n^2}{8}$.

### *Proof*

Consider a relation graph *R* and a species tree *S* for the MaxEC problem. Let $R'=(V(R), \emptyset )$ and $R''=\left(V(R), {V(R) \atopwithdelims ()2}\right)$ be two solutions for MaxES over instance *R* [for MaxEC over instance (*R*, *S*), respectively]. It is easy to see that $R'$ and $R''$ are both feasible solutions of MaxES and of MaxEC. Since for each $\{ u,v \}$, with $u,v \in V$, $u \ne v$, either one of $R'$ or $R''$ agrees with *R*, it holds

$$\begin{aligned} A(R,R')+ A(R,R'') = \left( {\begin{array}{c}n\\ 2\end{array}}\right) \end{aligned}$$

Then at least one of $R'$, $R''$ must have an agreement value of at least $$\frac{1}{2}\left( {\begin{array}{c}n\\ 2\end{array}}\right)$$, hence an optimal solution of MaxES and MaxEC has an agreement value of at least $$\frac{1}{2}\left( {\begin{array}{c}n\\ 2\end{array}}\right) \ge \frac{n^2}{8}$$. $\square$

Since it possible to compute an optimal solution of MaxES with additive cost $\varepsilon n^2$, for each $\varepsilon > 0$ [16], it follows that MaxES admits a PTAS.

Let *OPT*(*R*) be the value of an optimal solution on *R*, and let *c* be such that $OPT(R) = cn^2$. The additive $\varepsilon n^2$ approximation algorithm for cograph editing [16] yields a solution of value $(c - \varepsilon ) n^2$. As $c \ge 1{/}8$ by Lemma 6, $\varepsilon$ can be adjusted so that, for any $0< \varepsilon ' < 1$, $(c - \varepsilon )n^2 \ge (1 - \varepsilon ')cn^2$, hence yielding a PTAS. In the more general case, this algorithm does not ensure that genes from the same species remain paralogs. However, the authors of [16] claim that their approximation algorithm applies to any hereditary graph property (i.e. preserved after vertex-deletion), which holds for satisfiability.

### A PTAS for **MaxEC**

The PTAS for MaxES does not guarantee that the returned relation graph $R'$ (and its corresponding gene tree $G'$) is *S*-consistent with the given species tree *S*. In this section, we present a PTAS for MaxEC based on smooth-polynomial integer programming [22], a technique that has been applied to design PTAS for problems like maximum quartet consistency [23] or maximum consensus clustering [24].

As for maximum quartet consistency, the MaxEC problem is reduced to the assignment of leaves in ${\Gamma }$ to a tree, and the resulting tree is then used to to reconstruct a gene tree $G'$ that is consistent with *S* and whose relation graph requires at most $\varepsilon n^2$ modifications with respect to the original graph. In order to guarantee the *S*-consistency of the reconstructed gene tree, we need several technical arguments that are not used for maximum quartet consistency. Recall that we are considering binary trees.

Before giving the details, we present an overview of the PTAS. First, in “The compressed tree *G*^*k*^” section, we show that starting from a gene tree $G'$ we can compute a *compressed tree* $G^k$ that has at most *k* internal nodes and at most *k* leaves, where $k > 0$ is a constant. In order to construct such a compressed tree, first in “The unlabeled compressed tree *T*^*k*^” section we compute an *unlabeled compressed tree* $T^k$, and then in “A PTAS of MaxLA by smooth polynomial integer programming” section we compute a compressed tree $G^k$ from $T^k$ by using smooth-polynomial integer programming. Finally, we show in “Building a feasible solution” section how to reconstruct an *S*-consistent gene tree from $G^k$.

### The compressed tree $G^k$

First, we will focus on the compressed tree, and we show that, given an optimal solution $R'$ of MaxEC, there exists a compressed tree that respects a (large) subset of the speciation/duplication relations for $R'$.

**Fig. 3:**
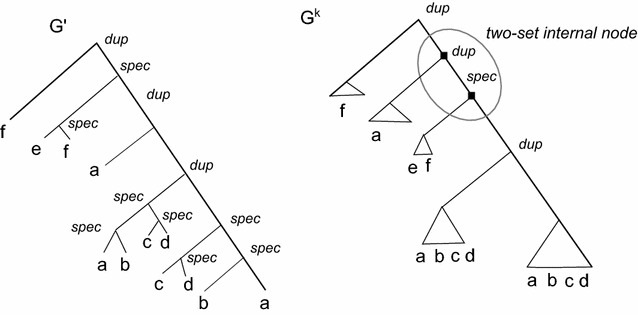
A compressed tree $G^k$ computed from a gene tree $G'$. Leaf-sets are represented with *triangles*

Consider an optimal solution $R'$ of MaxEC, and let $(G',ev_{G'})$ be a DS-tree, where $G'$ is the gene tree corresponding to $R'$. Recall that each internal node of $G'$ is associated by $ev_{G'}$ either with a duplication (*Dup*) or with a speciation (*Spec*). We present the formal definition of compressed tree $G^k$ associated with $(G',ev_{G'})$ (see Fig. 3).

#### **Definition 3**

Given a constant $k > 0$ and a DS-tree $(G',ev_{G'})$, a *compressed tree* $G^k$ associated with $(G',ev_{G'})$ is a tree that has at most *k* internal nodes and at most *k* leaves, which are called *leaf-sets*. An internal node *v* can be *a regular internal node* or can belong to a *two-set internal node* $\langle u,v \rangle$ such that $v \in ch(u)$, and both *u* and *v* have exactly one leaf-set as a child. The two-set internal nodes of $G^k$ are disjoint, that is $\langle u,v \rangle$ and $\langle v,w \rangle$ cannot be two-set internal nodes of $G^k$. Moreover, the following properties hold:

- the leaf-sets of $G^k$ induce a partition of ${\Gamma }$ and each leaf-set contains at most 8*n* / *k* elements of ${\Gamma }$
- each internal node of $G^k$ is associated with two possible events, *Dup* or *Spec*, by the function $ev_{G^k}$
- let $I_{v_1}$, $I_{v_2}$ be two leaf-sets connected to nodes $u_1$ and $u_2$, respectively, such that $\langle u_1,u_2 \rangle$ is not a two-set internal node, let $l_1 \in I_{v_1}$, $l_2 \in I_{v_2}$, and $x=lca_{G'}(l_1,l_2)$ and $y=ev_{G^k}(lca_{G^k}(I_{v_1},I_{v_2}))$, then $ev_{G'}(x) = ev_{G^k}(y)$.

Note that a leaf-set $I_v$ of $G^k$ is both a set of leaves of $G'$, and a leaf of $G^k$. It will sometimes be useful to clarify which one we wish to refer to, and so we denote by $L(I_v)$ the set of leaves that belong to $I_v$.

Now, we provide a constructive proof that shows that, starting from a solution $R'$ (whose corresponding gene tree is $G'$) of MaxEC over instance (*R*, *S*), there exists such a compressed tree $G^k$.

Consider the following algorithm. First, the algorithm initializes $G^k$ to $G'$ and all internal nodes are unmarked. Then, the algorithm traverses $G'$ and construct the tree $G^k$ as described in Algorithm Compressed Tree ($G'$).

When the algorithm stops it follows that each leaf-set has size at most 8*n* / *k*. Notice that, given a two-set internal node $\langle v_1,v_2 \rangle$, the leaves assigned to the leaf-sets $I_{z_1}$, $I_{z_2}$ connected to $v_1$ and $v_2$ are considered as a single leaf-set with reference to the relation between elements in $L(I_{z_1}) \cup L(I_{z_2})$.

Next, we show that the algorithm returns a compressed tree $G^k$, with at most *k* internals node and *k* leaf-sets.

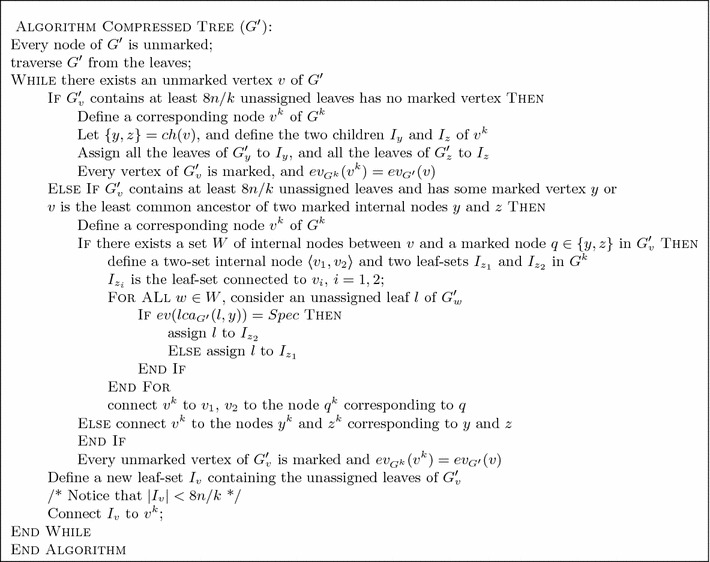



#### **Lemma 7**

*Given a gene tree* $G'$ *, Algorithm* Algorithm Compressed Tree ($G'$ *) returns a tree* $G^k$ *, with at most k internal nodes and k leaf-sets.*

#### *Proof*

First, consider the set of regular nodes of $G^k$. Consider the set $V^k_1$ of those nodes of $G^k$ that the algorithm defines because the subtree rooted at one of such nodes contains at least 8*n* / *k* unassigned leaves. It follows that at most *k* / 8 such nodes are chosen.

Consider the set $V^k_2$ of nodes of $G^k$ defined as internal nodes because they are the least common ancestor of two internal nodes of $V^k_1$. Now, if we restrict $G^k$ to $V^k_1 \cup V^k_2$, we obtain a tree having at most *k* / 8 leaves, as the leaves by construction are only nodes in $V^k_1$, where each internal node, except for the root, has degree at least three. Hence $|V^k_2| \le |V^k_1| \le k{/}8$.

Let *v* and *z* be two nodes in $V^k_1 \cup V^k_2$, such that *z* is an ancestor of *v* in $G^k$, and there are no other ancestor of *v* in $G^k$ that belongs to $V^k_1 \cup V^k_2$. It follows that, by construction, at most one two-set internal node on the path between *v* and *z* is defined in $G^k$. Hence at most two internal nodes are defined on the path between *v* and *z* in $G^k$, and since $|V^k_1 \cup V^k_2| \le k{/}4$, it follows that $G^k$ contains at most *k* / 4 two-set internal nodes. Thus $G^k$ consists of at most $k/4+k/2=(3/4) k$ internal nodes.

Now, consider the defined leaf-sets. For each two-set internal node $\langle v_1, v_2 \rangle$, there exists at most two leaf-sets connected with one of $\langle v_1, v_2 \rangle$, hence at most *k* / 2 leaf-sets. For each of the *k* / 4 internal node $v \in V^k_1 \cup V^k_2$, the leaves assigned to leaf-set connected to *v* are at most two, as $G'$ is binary. Hence there exists hence at most *k* / 2 leaf-sets connected to internal nodes of $v \in V^k_1 \cup V^k_2$. Hence, the number of leaf-set is bounded by $k/2+k/2=k$. $\square$

In order to prove that $G^k$ is a compressed tree, in addition to Lemma 7 we need the following result.

#### **Lemma 8**

*Given a gene tree* $G'$ *and a species tree S, let* $G^k$ *be the tree computed by Algorithm* Algorithm Compressed Tree ($G'$ *). Given two distinct leaf-sets* $I_u$ *and* $I_w$ *of* $G^k$ *connected to the internal nodes* *z* *and v, such that* $\langle z,v \rangle$ *is not a two-set internal node, let* $l_1 \in L(I_u)$ *and* $l_2 \in L(I_w)$ *. Let* $x^k =lca_{G^k}(I_u,I_w)$ *and* $x = lca_{G'}(l_1,l_2)$ *. Then* $ev_{G^k}(x^k) = ev_{G'}(x)$.

#### *Proof*

Let $lca_{G^k}(I_u,I_w) = x^k$. Assume that $I_u$ and $I_w$ are connected to the same internal node of $G^k$ (which must be $x^k$). Then when $x^k$ is defined by Algorithm Algorithm Compressed Tree ($G'$), its event is the same as the corresponding node *x* of $G'$. Assume that $I_u$ and $I_w$ are connected to different internal nodes of $G^k$, $u^k$ and $w^k$, respectively, corresponding to node *u* and *w* of $G'$. Consider $x=lca_{G'}(u,w)$ then Algorithm Algorithm Compressed Tree ($G'$) defines a corresponding node $x^k$ in $G^k$ such that $ev_{G^k}(x^k) = ev_{G'}(x)$.

Assume that $x^k$ belongs to a two-set internal node $\langle z,v \rangle$. Then, by construction, exactly one of $I_u$, $I_w$ (w.l.o.g. $I_u$) must be a leaf-set which is a child of $x^k$, and exactly one of $I_u$, $I_w$ (w.l.o.g. $I_w$) is a leaf-set connected to a strict descendant *c* of $x^k$, such that $c\ne z,v$. Let $y = lca_{G'}(l_1,l_2)$, for a leaf $l_1$ in $L(I_u)$ and a leaf $l_2$ in $L(I_w)$. By construction, $l_1 \in I_u$ only if $ev_{G^k}(x^k)=ev_{G'}(y)$, thus concluding the proof. $\square$

Lemmas 7 and 8 implies that Algorithm Algorithm Compressed Tree ($G'$) constructs a compressed gene tree $G^k$, as by construction the leaf-sets induce a partition of ${\Gamma }$.

Next, we show a lower bound on the agreement value of an optimal assignment of leaves to the leaf-sets $I_v$. We denote by $A(G^k,R)$ (the agreement between *R* and $G^k$) as the agreement for each pair of leaves $l_1, l_2 \in {\Gamma }$ that belong to two distinct leaf-sets $I_u$ and $I_w$ of $G^k$ connected to the internal nodes *u* and *v*, such that $\langle u,v \rangle$ is not a two-set internal node (notice that *u* and *v* may be the same node).

#### **Lemma 9**

*Given an optimal solution* $G^*$ *of* *MaxEC* *over instance (R, S) and a constant* $k>0$ *, let* $G^k$ *be the compressed tree computed starting from* $G^*$ *. Then* $A(G^k,R) \ge A(G^*,R)- \frac{64n^2}{k}$.

#### *Proof*

Consider an optimal solution $G^*$ of MaxEC over instance (*R*, *S*) and the compressed tree $G^k$ constructed from $G^*$. From Lemma 8, the pairs of leaves that belong to different leaf-sets (not connected to the same two-set internal node) have the same relations in $G^*$ and in $G^k$.

Consider the leaves of a same leaf-set $I_v$ or of two leaf-sets $I_w$ and $I_u$ which are connected to the same two-set internal node. Since $|I_v| \le \frac{8n}{k}$ and $|I_w \cup I_u| \le \frac{8n}{k}$, the number of relations between two leaves belonging to a common leaf-set is at most $\frac{64n^2}{k^2}$. Since there are at most *k* leaf-sets, the overall number of relations between pairs of leaves in $G^k$ with respect to $G^*$ are at most $\frac{64n^2}{k}$, hence $A(G^k,R) \ge A(G^*,R) - \frac{64n^2}{k}$. $\square$

### The unlabeled compressed tree $T^k$

The tree $G^k$ described above is of course not known, and it needs to be found. In this subsection we introduce the unlabeled compressed tree $T^k$ that is used to construct the compressed tree $G^k$. An *unlabeled compressed tree* $T^k$ is a compressed tree whose leaf-sets are empty. Here we introduce some properties of $T^k$ and we reduce the MaxEC problem to a second problem, called MaxLA (to be defined later). The PTAS iterates through the possible unlabeled compressed trees $T^k$. In particular, the PTAS iterates through (1) the structure of $T^k$, (2) the events associated with internal nodes of $T^k$, and (3) a set of labels that are allowed to be assigned to a leaf-set.

First, consider the structure of $T^k$. Since by Lemma 7 $G^k$ consists of at most *k* internal nodes and *k* leaf-sets, it follows that there are at most $2^{4k^2}$ possible topologies for the unlabeled compressed tree $T^k$. Indeed, the adjacency matrix of $T^k$ has size $4k^2$, and the possible adjacency matrices are at most $2^{4k^2}$. Moreover, for each topology, we define in time $O(2^k)$ the two-set internal nodes of $T^k$.

Now, consider the events associated with the internal nodes of $T^k$. For each unlabeled compressed tree $T^k$, the events associated with the internal nodes of $T^k$ are at most $2^k$ (two possible cases, *Dup* or *Spec*, for each of the *k* internal nodes). Overall we iterate though $O(2^{4k^2})$ possible unlabeled compressed tree $T^k$.

Consider now an unlabeled compressed tree $T^k$. In order to ensure that the gene tree $G'$ constructed from $T^k$ is *S*-consistent with the given species tree *S*, we must ensure that the speciation nodes of $G'$ are consistent with *S*. We define a mapping $s_{T^k}$ of the nodes of $T^k$, except the leaf-nodes connected to two-set internal nodes, to the nodes of *S* so that the mapping is *feasible*, that is the following conditions hold:

- if *v* is an ancestor of *u* in $T^k$, then $s_{T^k}(v)$ is an ancestor (not necessarily proper) of $s_{T^k}(u)$
- if *v* is an ancestor of *u* in $T^k$ and $ev_{T^k}(v)=Spec$, then $s_{T^k}(v)$ is a proper ancestor of $s_{T^k}(u)$

Based on the mapping $s_{T^k}$, define for each leaf-set $I_v$, the allowed set $A(I_v)$ of labels that can be assigned to a leaf-set $I_v$. If $I_v$ is a leaf-set not connected to a two-set internal node:

$$\begin{aligned} A(I_v)=\{ l{:}l \in {\mathcal{L}} (S_x) {\text { with }} x = s_{T^k}(I_v) \} \end{aligned}$$

If $I_v$ is a leaf-set connected to an internal node *u*, with $\langle u,w \rangle$ a two-set internal node (recall that $ev_{T^k}(u)=Dup$):

$$\begin{aligned} A(I_v)=\{ l: l \in {\mathcal{L}} (S_x) {\text { with }} x = s_{T^k}(u) \} \end{aligned}$$

If $I_v$ is a leaf-set connected to a two-set internal node *u*, with $\langle w,u \rangle$ a two-set internal node (recall that $ev_{T^k}(u)=Spec$), such that *z* is the only child of *u* in $T^k$ which is an internal node:

$$\begin{aligned} A(I_v)=\{ l: l \in {\mathcal{L}} (S_x) \setminus L(S_y), {\text { with }} x = s_{T^k}(u) {\text { and }} y = s_{T^k}(z) \} \end{aligned}$$

Since $T^k$ contains at most 2*k* nodes, the set of the feasible mappings $s_{T^k}$ are at most $O(n^{2k})$. Moreover, once the mapping $s_{T^k}$ is computed, $A(I_v)$ can be computed in *O*(*nk*) time.

Finally, for each set leaf-set $I_v$, we assign one leaf (denoted by $P(I_v)$) of $A(I_v)$ to $I_v$, in time $O(n^k)$. These leaves are called *preassigned leaves* and are assigned such that for each internal node *x* of $T^k$, the lca mapping of the preassigned leaves maps *x* to a node *y* of *S* such that $y=s_{T^k}(x)$. Notice that, given an optimal solution of MaxEC,there exists a feasible mapping with associated $A(I_v)$ and $P(I_v)$.

Now, we a able to define the MaxLA problem we will solve to compute the PTAS.

#### Maximum leaf assignment: (**MaxLA**)

Input: an unlabeled compressed tree $T^k$ with a feasible mapping $s_{T^k}$, a set of preassigned leaves $P(I_v)$, and a set $A(I_v)$, for each leaf-set $I_v$, a set ${\Gamma }$, a relation graph *R*, a specie tree *S*;
Output: a compressed tree $G^k$ obtained from $T^k$ by assigning leaves of ${\Gamma }$ to the leaf-set of $T^k$, where for each leaf-set $I_v$ only leaves of $A(I_v)$ are assigned to $I_v$, such that, $A(G^k,R)$ is maximized and each speciation node of $G^k$ is *S*-consistent.

By Lemma 9, it follows that an optimal solution of MaxLA has a an agreement value of at least $A(G^*,R)- \frac{64n^2}{k}$, where $G^*$ is the optimal solution of MaxEC.

### A PTAS of **MaxLA** by smooth polynomial integer programming

Now, we present a PTAS for MaxLA. Consider an unlabeled compressed tree $T^k$, with the corresponding allowed sets $A(I_v)$ and preassigned leaves $P(I_v)$. We start by introducing the smooth polynomial integer programming technique [22].

A polynomial having degree *c* is called *q*-smooth, for a constant $q>0$, if the coefficients of each degree-$\ell$ monomial belongs to the interval $[-qn^{c -\ell }, qn^{c -\ell }]$, for each $\ell$ with $1 \le \ell \le c$.

First, we define some constants:

- given a leaf-set $I_v$ of $T^k$ and ${\ell }\in {\Gamma }$, $a(I_v,l)=1$ if $l \in A(I_v)$ and 0 otherwise
- given two leaf-sets $I_v$, $I_w$ of $T^k$, $r(I_v,I_w)$ is equal to 1 if $lca_{T^k}(I_v,I_w)$ is a speciation, else (if $lca_{T^k}(I_v,I_w)$ is a duplication) $r(I_v,I_w)$ is equal to 0
- given two leaf-sets $I_v$, $I_w$ of $T^k$, $t(I_v,I_w)$ is a constant equal to 0 if $I_v$ and $I_w$ are connected to the same two-set internal node, else it is equal to 1
- given $l_1, l_2 \in {\Gamma }$, $e(l_1,l_2)=1$ if $l_1 l_2 \in E(R)$ and $e(l_1,l_2)=0$ otherwise

For each leaf-set $I_v$ of $T^k$ and each leaf $l \in {\Gamma }$, define a variable $x_{I_v,l}$ that has value 1 if *l* is assigned to $I_v$, else is 0 (notice that $x_{I_v,l}=1$ if *l* is a leaf preassigned to $I_v$). Given $l_1, l_2 \in {\Gamma }$, define

$$\begin{aligned} p(l_1,l_2)&= \sum _{I_v \ne I_w} x_{I_v,l_1} a(I_v,l_1) x_{I_w,l_2} a(I_w,l_2) r(I_v,I_w) e(l_1,l_2) t(I_v,I_w)\\ &\quad+ x_{I_v,l_1} a(I_v,l)x_{I_w,l_2} a(I_w,l_2)(1-r(I_v,I_w)) (1-e(l_1,l_2))t(I_v,I_w)\end{aligned}$$

Now, assume that $x_{I_v,l_1}=1$ and $x_{I_w,l_2}=1$, where $l_1 \in A(I_v)$, $l_2 \in A(I_w)$, $l_1$, $l_2$ do not belong to the same two-set internal node and $t(I_v,I_w)=1$; it holds that $p(l_1,l_2)=1$ if and only if (1) the lca of $I_v$ and $I_w$ is a speciation (hence $r(I_v,I_w)=1$) and $l_1$ and $l_2$ are connected by an edge in *R* (hence $e(l_1,l_2)=1$) or (2) the lca of $I_v$ and $I_w$ is a duplication (hence $r(I_v,I_w)=0$) and there is no edge between $l_1$ and $l_2$ in *R* (hence $e(l_1,l_2)=0$).

Finally define *p*(*x*) as follows:

$$\begin{aligned} p(x)= \sum _{l_1,l_2 \in L} p(l_1,l_2) \end{aligned}$$

The polynomial integer programming is defined as follows

$$\begin{aligned} p(x) {\text { is maximixed}} \end{aligned}$$

$$\begin{aligned} \sum _{v} x_{I_v,l}=1\quad \forall l\in L \end{aligned}$$

$$\begin{aligned} \sum _{l} x_{I_v,l} \le 8n/k \end{aligned}$$

The polynomial *p*(*x*) is 1-smooth.

Consider a solution for the smooth polynomial integer programming, given the correct unlabeled compressed tree $T^k$, the correct allowed sets $A(I_v)$ and the correct sets of preassigned leaves $P(I_v)$. For each $\varepsilon$, there is a polynomial time algorithm that produces a 0–1 assignment *x* to the leafset of $T^k$ (hence a compressed tree $G^k$), such that $p(x)\ge OPT - \varepsilon n^2$, where *OPT* is the maximum value of the smooth polynomial integer programming [22, 23].

Now, consider the labels assigned to different sets $I_{v}$. By Lemma 9, we have that the agreement between $G^k$ and *R* is at least $\frac{n^2}{8} - \frac{64n^2}{k}$. By Lemma 6, $A(G^*,R) \ge \frac{n^2}{8}$, where $G^*$ is an optimal solution of MaxEC, hence it holds

$$\begin{aligned} A(G^k,R) \ge A(G^*,R) - \varepsilon n^2 - \frac{64n^2}{k}= A(G^*,R) \left( 1- \frac{\varepsilon }{c} - \frac{1}{ck}\right) \end{aligned}$$

for a constant $c \ge 0$. By choosing $\varepsilon$ sufficiently small, and *k* sufficiently large, the PTAS for MaxLA follows.

Now, what we have to show is that, starting from a solution $G^k$ of MaxLA, it is possible to construct in polynomial time a gene tree $G'$ such that $G'$ is *S*-consistent and it has an agreement value not smaller than that of $G^k$.

### Building a feasible solution

Consider a compressed tree $G^k$ returned by the smooth polynomial integer programming. Next we show how to reconstruct a gene tree $G'$ which is consistent with *S*.

First, we consider only the set ${\Gamma }'$ of leaves $l \in {\Gamma }$ that are assigned to a leaf-set $I_v$, with $l \in A(I_v)$. Notice indeed that if a leaf is assigned to a leaf-set $I_v$ with $l \notin A(I_v)$, then it will give a contribution 0 in the smooth polynomial integer program, as $a(I_v,l_1)=0$, hence $p(l_1,l_2)=0$, for each other leaf in $l_2 \in {\Gamma }$. In this case, we construct a gene tree $G'$ only for the set of leaves ${\Gamma }'$, then we construct a new gene tree $G^*$ by joining $G'$ and a subtree $G''$ over leafset ${\Gamma }\setminus {\Gamma }'$ such that the internal nodes of $G''$ and the root of $G^*$ are all associated with a duplication.

**Fig. 4:**
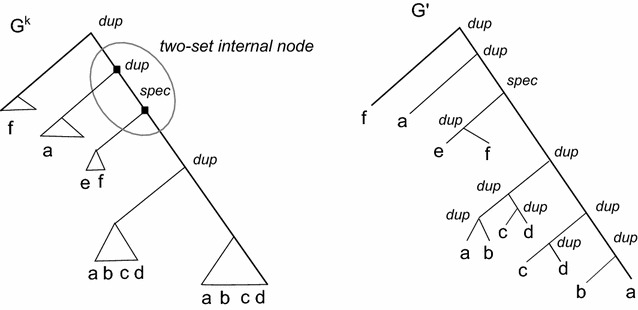
A compressed tree $G^k$ and the gene tree $G'$ computed starting from $G^k$

We focus now on the set of labels ${\Gamma }'$ and assume that no leaf *l* is assigned to a leaf-set $I_v$ such that $l \notin A(I_v)$. Starting from $G^k$ we construct in polynomial time the corresponding gene tree $G'$. $G'$ is computed by replacing each leaf-set $I_v$ of $G^k$ with a subtree labeled by the set $L(I_v)$ of leaves that belong to $I_v$ (see Fig. 4).

Consider the tree $G^k$, a leaf set $I_z$ of $G^k$ connected to a node *u* of $G^k$ and the set $L(I_z)$ of leaves assigned to $I_z$. We replace $I_z$ by a subtree $T'$ isomorphic to $S|L(I_z)$; each internal node of $T'$ is labeled as *Dup*. Notice that the root of $T'$ is connected to *u*.

As a last step, if $d>1$ copies of a label *l* belongs to a leaf set $I_v$, then we construct a subtree with *d* leaves all labeled by *l*, whose internal nodes are all associated with duplications.

We prove that the gene tree $G'$ constructed is *S*-consistent.

#### **Lemma 10**

*The tree* $G'$ *computed starting from* $G^k$ *is S-consistent.*

#### *Proof*

In order to ensure the *S*-consistency of $G'$, we must prove that for each node $v'$ of $G'$ with $ev_{G'}(v')=Spec$, each child of $v'$ is mapped to a proper descendant of $s_{G'}(v')$.

Consider a node $v'$ of $G'$ corresponding to an internal node *v* of $G^k$ such that $ev_{G'}(v')=Spec$ and $ev_{G^k}(v)=Spec$ and *v* is not part of a two-set internal node. We claim that $v'$ represents a speciation with respect to the species tree *S*. Let $ch(v')$ be the set of children of $v'$. Assume that $s_{G'}(v') = x'$, and that $s_{G'}(w') = x$, for some $w' \in ch(v')$. We show that *x* is a proper descendant of $x'$. Assume to the contrary that *x* and $x'$ are the same node. We claim that there exists a leaf *l* that is assigned to $I_z$, with $l \notin A(I_z)$, for some leaf-set of $G^k_{w}$, where *w* is the node of $G^k$ corresponding to $w'$. If the claim holds, then by construction $a(I_z,l)=0$ and this would contradict our earlier remark on such nodes not belonging to $\Gamma '$.

Hence, we must prove the claim: if *x* and $x'$ are the same node of *S*, then there exists a leaf $l \in {\Gamma }$ and a leaf-set $I_z$ in $G^k_w$, such that *l* is assigned to $I_z$, with $l \notin A(I_z)$. Assume that this is not the case. Since *v* is a speciation in $G^k$, it follows that the preassigned leaves define a mapping $s_{G^k}$ of *v* and *w* in two different nodes of *S*. Let $s_{G_k}(w)=y$, where *y* is a proper descendant of $x'$. Since $s_{G'}(v') = s_{G'}(w')$, it follows that there exists a leaf *l* of ${\Gamma }$ not in ${\mathcal{L}} (S_{z})$ that is assigned to a leaf-set $I_z$ in $G^k_{w}$, otherwise $w'$ would be mapped in *y*. Hence the claim holds.

Consider now the case that *v* belongs to a two-set internal node $\langle u,v \rangle$. Since $\langle u,v \rangle$ is a two-set internal node, $ev_{G^k}(v)=Spec$ and $ev_{G^k}(u)=Dup$. Moreover, let $I_z$ be the leafset connected to *v*. Let $z'$ be the root of the subtree of $G'$ isomorphic to $S|L(I_z)$ that replaced the $I_z$ leaf-set. Note that $z'$ is a child of $v'$. Let $q'$ be the other child of $v'$, and let *q* be the node of $G^k$ corresponding to $q'$.

Similarly to the previous case if $s_{G'}(v') = s_{G'}(z')$ or $s_{G'}(v') = s_{G'}(q')$, then we claim that there exists a leaf *l* that is assigned to $I_w$ with $l \notin A(I_w)$ for either $I_w = I_z$ or for some leaf-set $I_w$ in $G^k_q$. In order to prove the claim, first notice that, by definition, the set $A(I_z)$ contains only leaves of $L(S_x) \setminus L(S_y)$, where *x* and *y* are the nodes of *S* where *v* and *q* are mapped by $s_{G^k}$. Therefore, if $s_{G'}(z')$ is not a proper descendant of *x*, there must be a leaf $l \notin A(I_z)$ assigned to $I_z$. Similarly, if $s_{G'}(q')$ is not a proper descendant of *x*, because $s_{G^k}(q) = y$ is a proper descendant of *x*, there must be a leaf *l* assigned to $I_w$ in $G^k_q$ such that $l \notin A(I_w)$ (otherwise, $q'$ would be mapped to *y*). We can conclude that the lemma holds. $\square$

## Conclusion

We considered the minimization weighted and maximization unweighted variants of the problems of editing a relation graph for satisfiability and consistency. We provided complexity and algorithmic results for these variants. We showed that the problems that ask for the minimization of corrections on a weighted graph do not admit a constant factor approximation algorithm assuming the unique game conjecture and we gave an *n*-approximation algorithm, *n* being the number of vertices in the graph. We then provided polynomial time approximation schemes for the maximization variants of for unweighted graphs.

For future investigations, there are several interesting problems both from a theoretical and experimental point of view. First, from a theoretical point of view, it is open whether the minimization variant on unweighted graphs is approximable within constant factor or not. Moreover, another interesting direction would be to study whether it is possible to close the gap between the inapproximability result we have proved and the *n*-approximation algorithm.

From an experimental point of view, the main open problem is to test our approach to weighted graphs, and in particular to give a definition of weights that integrate those defined in different methods for orthology detection.

## References

[1] Ohno S (1970). Evolution by gene duplication.

[2] Goodman M, Czelusniak J, Moore GW, Romero-Herrera AE, Matsuda G (1979). Fitting the gene lineage into its species lineage, a parsimony strategy illustrated by cladograms constructed from globin sequences. Syst Zool..

[3] Tatusov RL, Galperin MY, Natale DA, Koonin EV (2000). The COG database: a tool for genome-scale analysis of protein functions. Nucl Acids Res..

[4] Li L, Stoeckert CJJ, Roos DS (2003). OrthoMCL: identification of ortholog groups for eukaryotic genomes. Genome Res..

[5] Berglund AC, Sjolund E, Ostlund G, Sonnhammer EL (2008). InParanoid 6: eukaryotic ortholog clusters with inparalogs. Nucl Acids Res.

[6] Lechner M, Findeiß S, Steiner L, Marz M, Stadler PF, Prohaska SJ (2011). Proteinortho: detection of co-orthologs in large-scale analysis. BMC Bioinf..

[7] Lafond M, Semeria M, Swenson KM, Tannier E, El-Mabrouk N (2013). Gene tree correction guided by orthology. BMC Bioinf.

[8] Lafond M, Swenson K, El-Mabrouk N, Chauve C, El Mabrouk N, Tannier E (2013). Error detection and correction of gene trees. Models and algorithms for genome evolution.

[9] Consortium TGO (2000). Gene ontology: tool for the unification of biology. Nat Genet..

[10] Lafond M, El-Mabrouk N (2014). Orthology and paralogy constraints: satisfiability and consistency. BMC Genomics.

[11] Hernandez-Rosales M, Hellmuth M, Wieseke N, Huber KT, Moulton V, Stadler PF (2012). From event-labeled gene trees to species trees. BMC Bioinf.

[12] Hellmuth M, Hernandez-Rosales M, Huber K, Moulton V, Stadler P, Wieseke N (2013). Orthology relations, symbolic ultrametrics, and cographs. J Math Biol..

[13] Hellmuth M, Wieseke N, Lechner M, Lenhof H-P, Middendorf M, Stadler PF (2014). Phylogenomics with paralogs. Proc Natl Acad Sci.

[14] Liu Y, Wang J, Guo J, Chen J (2012). Complexity and parameterized algorithms for cograph editing. Theor Comput Sci.

[15] Natanzon A, Shamir R, Sharan R (2001). Complexity classification of some edge modification problems. Discret Appl Math.

[16] Alon N, Stav U (2009). Hardness of edge-modification problems. Theor Comput Sci..

[17] Lafond M, Dondi R, El-Mabrouk N (2016). The link between orthology relations and gene trees: a correction perspective. Algorithms Mol Biol.

[18] Fitch WM (2000). Homology: a personal view on some of the problems. Trends Genet.

[19] Chawla S, Krauthgamer R, Kumar R, Rabani Y, Sivakumar D (2006). On the hardness of approximating multicut and sparsest-cut. Comput Complex.

[20] Corneil DG, Perl Y, Stewart LK (1985). A linear recognition algorithm for cographs. SIAM J Comput.

[21] Stoer M, Wagner F (1997). A simple min-cut algorithm. J ACM.

[22] Arora S, Frieze AM, Kaplan H (2002). A new rounding procedure for the assignment problem with applications to dense graph arrangement problems. Math Program.

[23] Jiang T, Kearney PE, Li M (2000). A polynomial time approximation scheme for inferring evolutionary trees from quartet topologies and its application. SIAM J Comput.

[24] Bonizzoni P, Vedova GD, Dondi R, Jiang T (2008). On the approximation of correlation clustering and consensus clustering. J Comput Syst Sci.

